# Traditional Chinese Medicine Intervenes in Vascular Dementia: Traditional Medicine Brings New Expectations

**DOI:** 10.3389/fphar.2021.689625

**Published:** 2021-06-14

**Authors:** Xue Bai, Meng Zhang

**Affiliations:** Department of Gerontology and Geriatrics, Shengjing Hospital of China Medical University, Shenyang, China

**Keywords:** vascular dementia, pathogenesis, traditional Chinese medicine, pharmacological mechanism, effective ingredients

## Abstract

Vascular dementia (VD) is one of the most common forms of dementia, referring to a group of symptoms that mainly manifest as advanced neurocognitive dysfunction induced by cerebrovascular disease (CVD). A significant number of studies have shown that traditional Chinese medicine (TCM) has a clinical impact on VD and thus has promising prospects. There have been many discussions regarding the pharmacological mechanisms involved in treatment of the kidney, elimination of turbidity, and promotion of blood circulation. TCM has a prominent effect on improving patients’ cognitive function and quality of life. In this review, we summarize the pathogenesis of VD in modern medicine and TCM, traditional prescriptions, single-agent effective ingredients and their pharmacological mechanisms for treating VD, highlight TCM’s characteristics, and discuss TCM’s multi-targeted mechanism for the treatment of VD.

## Introduction

As China’s population ages, the incidence rates of cerebrovascular disease (CVD) and vascular dementia (VD) rises year after year. In China, the total prevalence of dementia in people aged ≥ 65 years is 5.14–7.30%, implying that there are more than 8 million dementia patients, with VD being the most common form of non-degenerative dementia, accounting for 15–20% of dementia patients (Hugo and Ganguli, 2014). Fortunately, VD is a type of dementia that can be prevented and treated. The ideal medicine for VD should not only improve cognition but also improve production of vascular factors (Dong et al., 2019). However, chemical drugs cannot achieve such dual effects, while TCM has the advantage of targeting multiple targets or pathways for improving cognition and vascular factors. In recent years, an increasing number of studies have been carried out on the clinical efficacy and pharmacological effects of TCM on VD treatment.

Cognitive function is represented by advanced activities in the cerebral cortex and is closely related to the “mind and deity,” as described in TCM. VD is a diagnostic term used in modern medicine. Although there is no clear record in the ancient literature, terms like “idiot,” “dementia,” “foolishness,” and “spiritual dementia” have been used to express VD. The “Clinical Guideline Medical Record” recorded that “first stroke, left with a daze”, in relation to understanding VD. “Shen Nong’s Materia Medica” reported that long-term use of *Shichangpu*, *Ginseng*, *Yuanzhi*, and *Ganoderma lucidum* may help one “not forget, not to be lost” and “benefit wisdom.” The etiology and pathogenesis of VD are complex (Dong et al., 2016). Dementia may be caused due to kidney deficiency, liver depression, phlegm turbidity, blood deficiency, or stasis, according to ancient medical doctors. Medical doctors of all generations believed the disease was asthenia in origin and superficiality. They believed VD was related to insufficiency of the kidney, a deficiency of Qi and blood in essence, stagnation of Qi, phlegm obstruction, and blood stasis. Therefore, nourishing the kidney and filling the essence, strengthening the spleen and Qi, dissipating phlegm for resuscitation, promoting blood circulation and stasis, calming the liver and Yang, while calming and nourishing the mind are modes of treating VD, according to TCM.

Modern medicine has discussed the possible pathogenesis of VD in several directions. Cerebral blood flow, intracranial artery stenosis, and decreased cerebral blood flow are related to the incidence of VD (Román et al., 2002). The Notch 3 gene, the apolipoprotein E gene, and polymorphism of the atrial natriuretic peptide gene are related to the pathogenesis of VD (Ikram et al., 2017). The following sections address the use of TCM in VD through various pathogenic routes.

## The Cholinergic System in VD

The central cholinergic system is closely related to learning and memory. Abnormal activity of acetylcholine transferase (ChAT) and dysregulation of acetylcholine (Ach) metabolism are important factors in central nervous system (CNS) aging, as well as reasons for learning and memory decline. Studies have shown that damage to central cholinergic neurons associated with chronic cerebral insufficiency may be the reason for the cognitive impairment in VD. This cholinergic neuronal injury further damages the hippocampal circuit associated with neurobiochemical basis for learning and memory in the brain (Cao et al., 2016). Patients with VD have substantially diminished central cholinergic nerve control, which is primarily manifested by decreased Ach activity. Ach is a neurotransmitter in the cholinergic pathway that participates in maintaining consciousness, learning, and memory. ChAT catalyzes Ach synthesis and its function in brain is closely related to learning and memory (Téglás et al., 2019). It is believed that a decrease in ChAT activity in VD patients' brain tissues leads to a decrease in Ach, which blocks the transmission of cholinergic nerve information, resulting in cognitive impairment (Zhu et al., 2020). Hence, improving the function of the cholinergic system is one of the main methods to prevent and treat VD.

Gastrodia elata pill, a proprietary Chinese medicine, is a damp clearing agent that relieves wind and dehumidification, soothes collaterals and analgesia, and tones the liver and kidney. The Gastrodia elata pill inhibits AChE activity and improves learning ability in VD rats (Li et al., 2008). Wu et al. reported that the Shenmayizhi decoction (SMYZ) significantly improved behavioral performance in VD rats by increasing the ChAT levels while decreasing the AChE level. In addition, an increased level of the acetylcholine M1 receptor was found after treatment with the SMYZ decoction, suggesting that SMYZ mitigates cognitive deficits by preventing cholinergic system dysfunction (Wu et al., 2019b). Shengjiang powder is an ancient prescription from “Shangshu Quanshu.” This powder has been used to treat blockage of the lung, Yin and Yang disorders, and unconsciousness (Qian et al., 2019). Studies have shown that Shengjiang powder improves cognitive dysfunction in VD rats by increasing the Ach levels in the hippocampus and reducing AchE activity (Ge et al., 2015). The Qufeng Tongluo Prescription originated from “Qian Jia Miao Fang.” This medicine dispels the wind, reduces phlegm, relaxes collaterals, and nourishes the blood and liver (Wu et al., 2013). Qufeng Tongluo significantly increases ChAT expression in a VD rat model, which improves cholinergic system function and memory in the VD rat model (Park et al., 2009). The Yuan-Zhi Decoction (YZD) is from the “Yixin Prescription.” It is mainly used to treat stroke, restlessness, convulsions, speech errors, trance, confusion, and tinnitus (Liu et al., 2020b). YZD also upregulates ChAT expression in the cholinergic neural circuit of VD mice, which improves learning and memory (Wang et al., 2020c). In brief, a variety of TCM prescriptions can improve the cognitive function of VD patients by regulating the function of the central cholinergic system.

## Amyloid β and Hyperphosphorylated TAU Protein in VD

Previous studies have suggested that neurofibrillary tangles (NFT) and amyloid β (Aβ) are involved in the pathogenesis of VD. Aβ deposition directly causes VD by aggravating the progress of cerebral amyloid angiopathy, and reducing blood flow by enhancing vasoconstriction, thereby causing or aggravating the occurrence and development of VD (Ecay-Torres et al., 2018). Tau hyperphosphorylation occurs in neurons, forming tangles of nerve fibers and reducing binding power with tubulin. In addition, tubulin polymerization and stability of the microtubules are lost. Damage to the microtubular structure and axon transport, as well as loss of synapses, leads to nerve cells dysfunction, neuronal degeneration and cognitive dysfunction. Studies have shown that aggregations of abnormally phosphorylated Tau protein (P-tau) are highly correlated with VD (Faraco et al., 2019). Therefore, interventions to prevent Aβ aggregation and abnormal phosphorylation of the Tau protein are some of the main measures to prevent VD.

Li et al. reported that YZD significantly restored impaired cognitive function after bilateral common carotid artery occlusion in a rat model. Furthermore, YZD was found to decrease the levels of Aβ aggregates and autophagy-related proteins ATG5 and ATG12 in the hippocampus (Liu et al., 2020b). Yangxue Qingnao Granules (YXQNG) suppresses Aβ_42_ fibrillogenesis and alters the β-sheet conformation, indicating an inhibition of primary nucleation of the amyloid protein. The Qufeng Tongluo prescription significantly inhibits the abnormal P-tau protein in VD model rats while also reducing the expression of the Aβ protein and neuronal damage, thereby improving learning and memory in VD rats. In general, TCM could serve as therapeutic agent for the treatment of VD by reducing the level of Aβ aggregates and inhibiting the formation of abnormal P-tau protein.

## Oxidative Stress and Scavenging Free Radicals in VD

During cerebral ischemia and hypoxia, the unsaturated fatty acids on the neuron cell membrane are easily oxidized, producing large quantities of free radicals, causing a chain reaction that attacks the membrane structure of neighboring cells and triggers lipid peroxidation. After surpassing the major scavenging ability of superoxide dismutase (SOD) and glutathione peroxidase (GSH-Px), the free radicals accumulate abnormally leading to massive neuronal apoptosis or necrosis, which induces cognitive dysfunction (Du et al., 2018; Raz et al., 2019). Therefore, antagonizing oxidative stress may be helpful to prevent and treat VD.

Naokang Capsule (NKC) is a hospital preparation used by the Beijing Tibetan Hospital to treat CVD. It nourishes the kidney, invigorates the phlegm, and promotes blood circulation. Early studies show that NKC improves brain cell metabolism and promotes brain cell development. In addition, NKC enhances SOD activity in VD rats and reduces free radical levels, thereby improving learning ability and memory in VD rats (Xiong et al., 2015). Shenmayizhi decoction (SMYZD) is composed of *Ginseng*, *Tianma*, *Shiyingpu*, and *Chuan Shao*. It has the function of tonifying deficiencies and increasing wisdom, calming the liver and quenching the wind, activating blood and removing fatigue, eliminating phlegm and opening the orifices. Wang et al. reported that SMYZD significantly improved the outcomes of Mini-Mental State Examination and “activities of daily living” scores in VD patients, suggesting that SMYZD is a safe and effective drug for treating VD (Wang et al., 2019b). The Guiqicongzhi Decoction protects brain tissues while improving the pathological damage and memory functions in VD rats by regulating the expression of heat shock protein (HSP) 27 and HSP70 (Wu et al., 2015a). Therefore, TCM could improve VD by enhancing SOD activity and reducing free radical levels. However, the current conclusions are mainly based on preclinical studies in animal models, and further clinical studies are needed to confirm the therapeutic mechanisms of TCM.

## Neuronal Apoptosis in VD

Neuronal apoptosis is an important form of cell death following cerebral ischemic injury, with *Cytc* and *Bax* being the genes most closely linked to apoptosis. A large number of studies have confirmed that neuronal apoptosis in the brain is the main pathogenesis of VD (Huo et al., 2019). The Bcl-2 gene family and the caspase family are the most widely studied proteins in the pathogenesis of VD (Ji et al., 2010). Zhao et al. demonstrated the ratio of Bax to Bcl-2 and the cleaved caspase-3 expression were significantly higher in VD rats than those in control groups, suggesting that severe apoptosis occurred via the mitochondria-related Bax/Bcl-2 and caspase-3 pathway in the progression of chronic cerebral hypoperfusion to VD (Zhao et al., 2018). Sun et al. also suggested that the expression levels of Bcl-2, Bax and Bcl-2/Bax ratio were significantly decreased in the VD rats compared to the normal control group (Sun et al., 2014). However, regulation of cleaved caspase-3, Bax, Bcl-2 levels could inhibit memory impairment in a VD rat model. Therefore, inhibiting neuronal apoptosis has become an important pathway to prevent and treat VD (Xu et al., 2019).

Yifei Xuanfei Jiangzhuo Formula (YXJF) has the effects of nourishing and expelling the lungs, clearing the organs, reducing turbidity, ameliorating memory impairments in rats with cerebral ischemia/reperfusion, inhibiting hippocampal apoptosis in a dose-dependent manner, attenuating the increase in protein expression of Bcl-2-associated X protein and c-Jun, and reducing Bcl-2 protein expression in the hippocampal tissue of the rats (Wu et al., 2015b). Modified Dioscorea Pills (MDP) promotes the regeneration and repair of hippocampal neurons by inhibiting downregulation of the ERK5/BMK1 signaling pathway, improving disorder in the hippocampal tissue structure in VD model rats, and reducing apoptosis in hippocampal neurons, thereby improving learning ability and memory (Li et al., 2020b). The Jiannao Yizhi Formula (JYF) is obtained from the “Therapeutic Materia Medica” and has the effect of nourishing the mind and strengthening the qi. JYF improves cognitive function of patients with Alzheimer’s disease (Wang et al., 2020a). It has also been identified as an effective drug for VD patients in clinically controlled studies and hence deserves further investigation and use (Zhang et al., 2002). Yang et al. found that JYF decreases the neuron apoptosis rate and Bax levels, increases Bcl-2 levels, and decreases the Bcl-2/Bax ratio in the SAMP 8 brain, which is probably part of the mechanism of inhibiting apoptosis and improving cognitive function (Yang et al., 2006), suggesting anti-neuronal apoptosis is also a promising therapeutic target for VD.

## Inflammatory Response in VD

Inflammatory cytokines involve in the pathogenesis VD. By stimulating the production of other cytokines and inflammatory mediators, inflammatory cytokines induce infiltration of white blood cells and increase the presence of glial cells. Studies have determined that inflammatory cytokines are involved in the neuropathic damage of VD (Tarkowski et al., 2001; Mulugeta et al., 2008). Since the inflammatory response has a particular role in neuronal degenerative diseases, inhibiting such responses could serve as a neuroprotective strategy (Voet et al., 2019). Therefore, it is of great significance to explore the role of inflammatory cytokines in the process of VD.

YXQN nourishes the blood, calms the liver, and promotes blood circulation. YXQN could inhibit activation and proliferation of microglia in the hippocampal CA1 region of VD rats (Li et al., 2016). Nao Tai Fang (NTF) increases the learning ability of VD rats by activating the SIRT1/NF-κBp65 signaling pathway, downregulating NF-κB, and upregulating IκBα expression (Liao et al., 2015). The Gui Qi Cong Zhi Decoction protects brain tissue and improves the memory functions of VD rats by regulating HSP27 and HSP70 expression (Wu et al., 2015a). Anti-inflammatory treatment is bound to be one of the most important targets of VD treatment, even though studies of TCM on inhibiting inflammatory response and inflammatory factors are minimal at this time. Given its important role in the pathogenesis of VD, anti-inflammatory treatment is bound to be one of the most important targets of VD treatment.

## Synaptic Plasticity in VD

It has been reported that the incidence of VD is significantly related with damage to synaptic plasticity (Dong et al., 2018). Studies identified a significant reduction in the number of synapses in the hippocampal CA1 area of VD rats relative to normal rats. The presynaptic, post-synaptic membrane, and intersynaptic space were blurred, and the dense substance was missing (Dong et al., 2018). Therefore, improving synaptic plasticity has become one of the main measures to prevent VD.

YXJF increases the expression of hippocampal CaMKP-Ⅱ and synapsin in VD rat brain tissue and improves learning (Iwasaki et al., 2012). MDP could increase the level of BDNF mRNA in the hippocampus of VD rats by increasing synaptic plasticity. In addition, MDP promotes proliferation of oligodendrocytes and axon myelination in VD rats, protects against synaptic damage and degeneration under ischemic conditions, promotes synaptic regeneration and reconstruction, ensures synaptic plasticity and accurate transmission of neural information, and thus could contribute to VD treatment (Wang et al., 2019a; Li et al., 2020b).

## Pharmacological Mechanisms of TCM for Cerebral Small Vessel Disease

Cerebral small vessel disease (CSVD) is a series of clinical, imaging and pathological syndesmosis caused by various causes affecting the small arteries, arterioles, capillaries, venules and venules in the brain (Quick et al., 2021). In China, lacunar infarction caused by CSVD accounts for 25–50% of ischemic stroke, higher than that in western countries (Tsai et al., 2013). The prevalence of high signal in white matter increased from 50 to 95% between 45 and 80 years of age (Wen et al., 2009). The prevalence of cerebral microhemorrhage was 24%, and increased gradually with age, 17.8% in people aged 60–69 years, and 38.8% in people aged ≥80 years (Pinter et al., 2015). CSVD is one of the most common causes of cognitive impairment, and the cognitive dysfunction caused by CSVD can account for 36–67% of VD (Wu et al., 2019a).

In TCM, cerebral small vessels are mostly collaterals (络脉). Collaterals are small and spread all over the body, and serve as important channels for Qi and blood to run in the human body. Collateral stasis due to multiple causes is known to cause collateral disease (络病). The clinical manifestations of collateral diseases are complex and diverse, mainly blood, phlegm, pain syndromes, and arthromyodynia, which can cause visceral dysfunction and structural damage (white matter lesions or CSVD lesions). The collaterals in the brain include Qi and blood-collateral, Qi-collateral has the function of dispersing menstrual Qi, whereas blood-collateral carries blood to circulate blood, so as to achieve the function of moisturizing and nourishing brain tissue. If spleen, stomach, and kidney deficiencies are present, Qi emptiness in the brain will occur, and the inability to promote the movement of Qi and blood in the collaterals will result in Qi-deficiency and blood stasis. If blood stasis blocks the collaterals for a long time, it will only further aggravate collateral diseases.

CSVD originates in the brain and is closely related to the heart, kidney, liver and spleen. It is an essential empty and out solid disease. Impairment of the tight junctions between cells and vascular endothelial dysfunction are caused by deficiency in right-Qi while Qi and blood are lost in movement. Plasma components enter the brain tissue through the damaged intercellular space and injure the white matter of the brain. Therefore, the basic treatment principle of CSVD is Fuzheng Quxie. On the one hand, Fuzheng can promote the metaplasia and normal operation of Qi, blood and body fluid; on the other hand, it can protect the vascular endothelial cells and prevent body fluid leakage and thrombosis.

A recent meta-analysis suggested that three traditional Chinese herbal medicine, Nao XinTong (NXT), Nao MaiTai (NMT) and Tong XinLuo (TXL) had the strongest evidence to justify further research for VD therapy (Chan et al., 2018). NXT is composed of sixteen TCMs such as *Astragalus*, *Danshen*, *Chuanxiong,* and *Safflower*. It functions by invigorating Qi and activating blood while improving the local blood supply. Modern pharmacological studies have shown that NXT can expand cerebral blood vessels, promote the establishment of collateral circulation, increase cerebral blood flow, protect vascular endothelial cells, and improve red blood cell deformability (Viswanathan et al., 2009). In addition, it can inhibit platelet aggregation, improve cerebral hypoxia, scavenge free radicals, and inhibit thrombosis (Escandon et al., 2010). The main components of NMT are *Salvia miltiorrhiza*, *Panax notoginseng*, *Red Ginseng* and *Ginkgo biloba*. Previous research has shown that NMT can reduce brain damage in VD rats, inhibit nerve cell apoptosis, and have anti-inflammatory and antioxidant effects (Huang et al., 2019). TXL is a traditional prescription composed of *Ginseng* and *Leeches* based on the theory of collateral disease. It can supplement Qi, activate blood circulation and refresh Tongqiao. Modern studies have shown that the pharmacological mechanisms of TXL include: 1) reduction of endothelin levels in plasma, upregulation of oxygen monoxide content in endothelial cells, and improvement of endothelial dysfunction and vasospasm (Dai et al., 2011); 2) reduction of thrombin activity to inhibit platelet agglutination, reduction of blood viscosity, and promotion of blood circulation (Liu et al., 2014); 3) regulation of vascular endothelial dysfunction, inhibition of platelet agglutination to promote the blood circulation of ischemic brain tissue, improvement of vascular reserve capacity and improvement of cognitive function (Yin et al., 2010; Fei et al., 2017).

## Analysis of the Classic TCM Prescription Characterstics

Many experimental studies have highlighted the potential advantages of TCM in preventing and treating VD. Due to the various etiologies and complex pathogenesis of VD, animal models cannot fully reproduce the pathological changes observed in human VD. In addition to the complexity of TCM, experimental evaluation standards have not been unified, and a complete system of rational and prescription medicines has not been formed. All of these problems have brought great challenges to prevent and treat VD using TCM. By reviewing the traditional prescriptions described here for treating VD by focusing on multi-target or pathways associated with TCM, we have increased our understanding of these traditional prescriptions.

**FIGURE 1:**
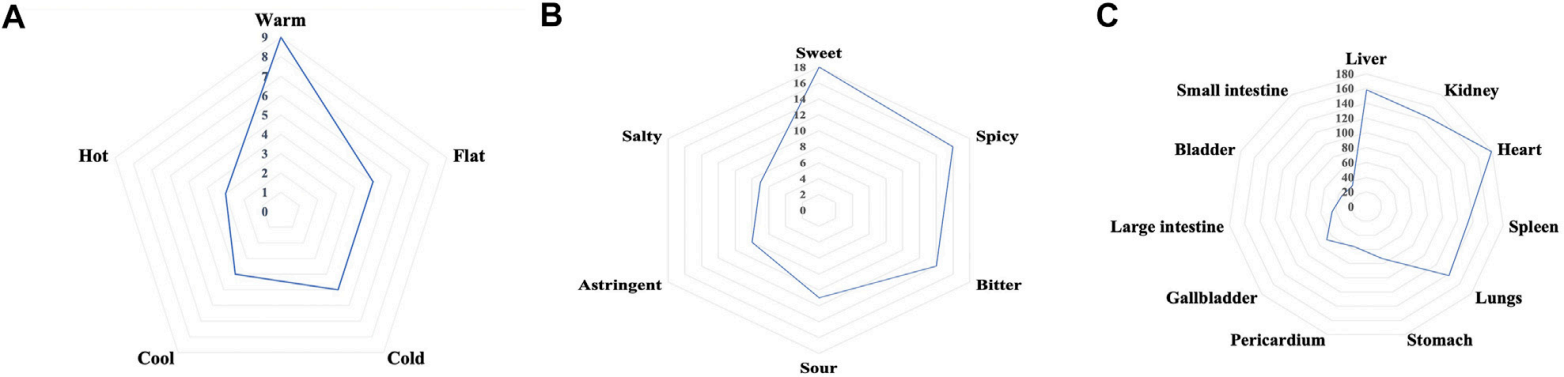
The distribution properties of **(A)**, flavors, **(B)** channels, and **(C)** frequencies of traditional Chinese medicine prescriptions for treating vascular dementia.

The properties, flavors, and channels of TCM prescriptions are the theoretical basis for explaining the effects of the medicine. We analyzed the properties and flavors of the above 20 TCMs, and the results showed that warm medicines were used the most, followed by flat and cold ones, and the cool and hot ones were used least. The sweet, spicy, and bitter flavors accounted for the largest proportion of medicine flavors, followed by sour, astringent, and salty. The distribution of the properties and flavors are shown in Figures 1A,B. TCM posits that VD is related to the five internal organs, the brain is the sea of the marrow, the kidney stores essence, and the main bone produces marrow. The brain marrow can be nourished, so that brain consciousness can be returned to normal; the liver master calms emotional feelings, encourages qi and blood circulation, and hence maintains the brain operation. The heart mainly controls the blood and provides the material basis and power for the functioning of the brain. The spleen is responsible for transport; it metabolizes sperm and blood, dredges stasis, and is used to nourish the brain and the five internal organs. The lungs are responsible for lowering and regulating the water channels, which prevent water, wet phlegm, and stagnation from remaining in the body, while assisting the other four organs to function. The liver, kidney, heart, spleen, and lungs account for the vast majority of the TCM channels, followed by the stomach, pericardium, gallbladder, large intestine, bladder, and small intestine. The distribution of channel frequencies is shown in Figure 1C.

## Intervention of Single MEDICINE and the Effectiveness of TCM for Treating VD

**FIGURE 2:**
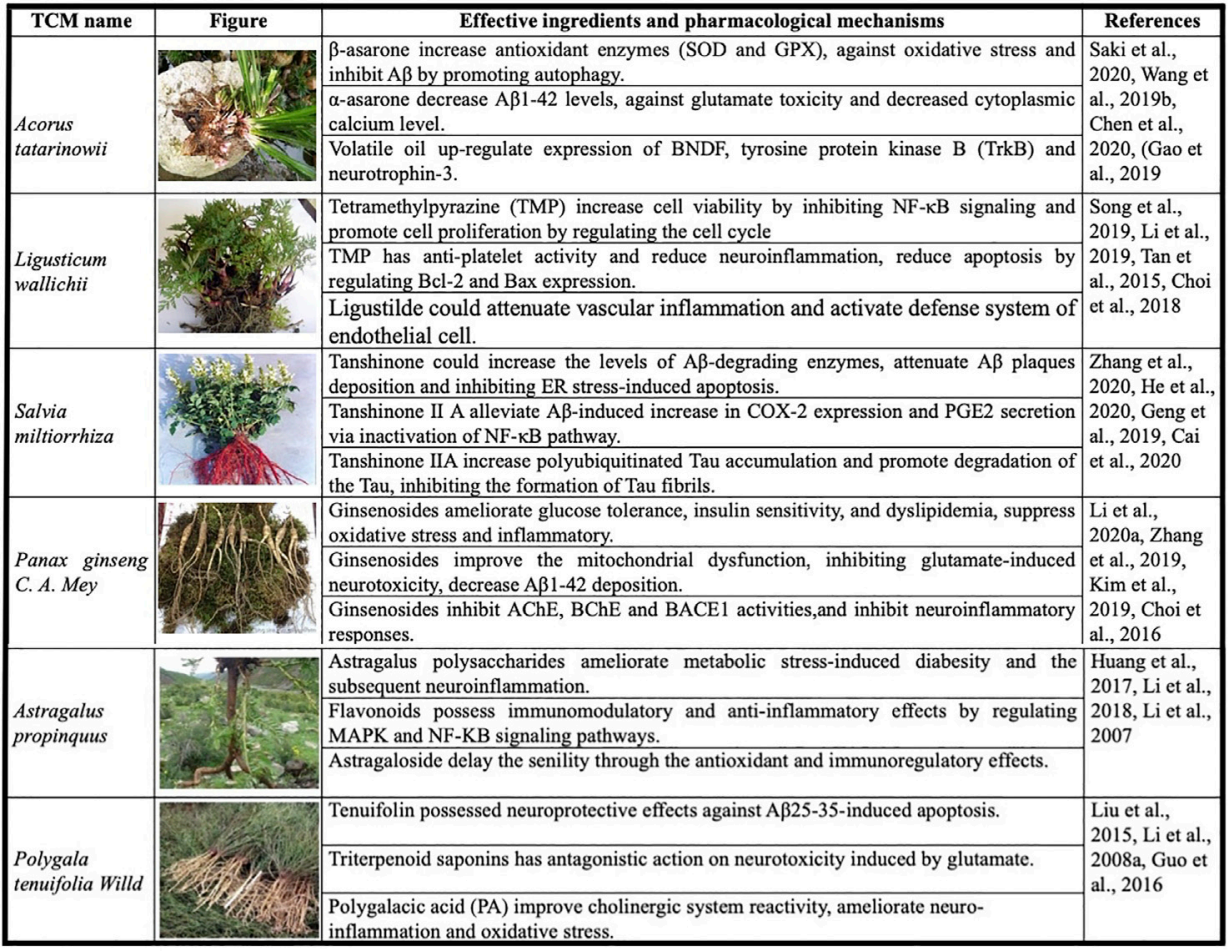
Most commonly used “single medicines” and the effective ingredients for vascular dementia.

Some “single medicines” appear frequently in these TCM classic prescriptions, implying that “single medicines” can play a role in VD care. Further, we’ll review studies on the most widely used “single medicines” and their ingredients that have been shown to be effective against VD. (Figure 2).

*Acorus tatarinowii* is an Araceae plant, originally recorded in the “Shennong Materia Medica.” It has warm, spicy, and bitter flavor properties. It has effects of opening phlegm, refreshing the mind and nourishing, dampening, and appetizing. *A. tatarinowii* is mainly used to treat dizziness, epilepsy, forgetfulness, insomnia, tinnitus, and deafness. *A. tatarinowii* mainly contains volatile ingredients, such as α-asarone, β-asarone, and methyl eugenol. Such ingredients have anti-VD and other pharmacological effects. Saki et al. reported that β-asarone significantly increases the levels of antioxidant enzymes, including SOD and GSH-Px. β-asarone is also effective for protecting against the oxidative stress and neuronal damage induced by Aβ (Saki et al., 2020). Chen et al. demonstrated that α-asarone improves spatial memory, reduces neuronal injury, and decreases Aβ_1–42_ levels in the hippocampus of aged rats. α-Asarone also has neuroprotective effects against glutamate toxicity and decreases the cytoplasmic calcium level in primary hippocampal neurons (Chen et al., 2020). Therefore, *A. gramineus* and its ingredients may have potential therapeutic effects on VD through multiple pathways.

*Ligusticum wallichii* is mainly distributed in Sichuan, China. It is a Chinese medicinal plant, commonly used to promote blood circulation and qi, dispel wind, and relieve pain. It has a spicy and warm fragrance along with wide range of functions such as promotion of blood circulation and removal of blood stasis. The effective ingredients in *L. wallichii* include tetramethylpyrazine (TMP), ligustilide, and wallichilide. TMP has anti-platelet activity by suppressing the Akt signaling pathway (Li et al., 2019), reducing neuroinflammation, and protecting neurons during cerebral ischemia/reperfusion injury (Tan et al., 2015). TMP also reduces apoptosis by regulating Bcl-2 and Bax expression thus rendering a protective effect against ischemia/reperfusion injury of the spinal cord (Fan et al., 2006). Choi et al. reported that ligustilide attenuates vascular inflammation and activates the defense system of endothelial cells (Choi et al., 2018). *L. wallichii* is a commonly used Chinese medicine to improve ischemic stroke by enhancing cerebral blood circulation. In addition to its anti-platelet aggregation and anti-thrombosis effects, it has anti-inflammatory and anti-apoptotic effects (Mazzio et al., 2017), suggesting that *ligusticum wallichii* may serve the therapeutic role in VD through anti-apoptotic and anti-vascular inflammation mechanisms.

*Salvia miltiorrhiza* is the dried root and rhizome of *S. miltiorrhiza* Bge*.* It has slightly cold and bitter properties and belongs to the heart and liver channel. *S. miltiorrhiza* promotes blood circulation, removing blood stasis, clears menstruation, and analgesia. The main effective ingredients of *S. miltiorrhiza* are tanshinone Ⅰ, Ⅱ A, Ⅱ B, and ferruginol. Current research on the active ingredients in *S. miltiorrhiza* has mainly focused on the tanshinone ⅡA mechanism. The tanshinone IIA could lower serum lipids levels, stabilize atherosclerotic plaques, and reduce endothelial injury and inflammatory damage by activating the TGF-β/PI3K/Akt/eNOS pathway (Wang et al., 2020b). In addition, it improves cell viability and protects against Aβ-induced apoptosis in a dose-dependent manner by increasing the levels of Aβ-degrading enzymes (Liu et al., 2020a; Zhang et al., 2020). Tanshinone Ⅱ A also improves memory by attenuating deposition of Aβ plaque and inhibiting ER stress-induced apoptosis (He et al., 2020). It is expected to become an effective drug for treating VD.

*Panax ginseng* C. A. Mey is a perennial herbaceous plant in the Umbelliferae family. Its flavors are sweet and slightly bitter. The properties are warm and calm. It has functions to replenish qi and solidify, regenerate, and soothe the nerves and the mind. The effective ingredients of *P. ginseng* are mainly ginsenosides A–F, and the main volatile oil ingredient is panaxen. Li et al. suggested that ginsenosides could improve cognitive dysfunction by ameliorating glucose tolerance, insulin sensitivity, and dyslipidemia, suppressing oxidative stress and the inflammatory response while modulating the NLRP3 inflammatory pathway and ER stress (Li et al., 2020a). Ginsenosides also prevent cognitive impairment in Alzheimer's disease rats by improving mitochondrial dysfunction (Zhang et al., 2019). Ginsenosides improve cognitive deficits and decrease Aβ_1-42_ deposition in the hippocampus of VD rats by enhancing the expression of pSer9-glycogen synthase kinase 3β and insulin degrading enzyme (Zong et al., 2019). In addition, Choi et al. suggested that ginsenosides have the potential to prevent dementia by inhibiting AChE, BChE, and BACE1 activities, as well as scavenging peroxynitrite and inhibiting the formation of nitrotyrosine (Choi et al., 2016). These studies confirm the therapeutic value of ginsenosides for treating VD based on different pharmacological mechanisms. Ginsenosides are expected to be in clinical trials as soon as possible to confirm their effectiveness in VD patients.

*Astragalus propinquus* is a leguminous plant that is mainly distributed in Russia and China. *A. propinquus* roots can be used as medicine; they are sweet in flavor, and warm in property. *A. propinquus* roots invigorates Qi and acts as a diuretic and cardiotonic. *A. propinquus* can be used to treat spontaneous sweating, internal injuries due to a deficiency of Qi. The main chemical components in *A. propinquus* are astragalus polysaccharides, flavonoids, and astragaloside. The pharmacological effects of *A. propinquus* include improved immune function and memory (Fu et al., 2014). Astragalus polysaccharides could ameliorate metabolic stress-induced diabetes and the subsequent neuroinflammation and improve behavioral performance in metabolically stressed transgenic mice (Huang et al., 2017). Flavonoids from *A. propinquus* have *in vivo* and *in vitro* immunomodulatory and anti-inflammatory effects (Li et al., 2018). Astragaloside may delay senility in aging rats induced by hydrocortisone through antioxidant and immunoregulatory effects (Li et al., 2007). Studies on applying the active ingredients of *A. propinquus* in VD are limited, but many active ingredients from *A. propinquus* may contribute immune regulation, which may lead to possible VD treatments and be worthy of further study.

*Polygala tenuifolia* Willd is primarily grown in Northeast China and Sichuan Province. It has the functions of calming the mind, removing phlegm, and reducing swelling. *P. tenuifolia* has been used for the treatment of insomnia, dreams, and forgetfulness. The main active ingredients in *P. tenuifolia* are saponins and ketones. The identified structures are the *Polygala* saponins A, B, E, F, and G. Tenuifolin, a secondary saponin from a hydrolysate of polygalasaponins, possesses neuroprotective effects against Aβ_25–35_-induced apoptosis in PC12 cells, and significantly improves the cognitive impairment induced by an intrahippocampal injection of Aβ_25–35_ in mice. Thus, Liu et al. suggested that tenuifolin is one of the active constituents in *P. tenuifolia* that works against the neurotoxicity induced by the Aβ_25–35_ peptide *in vitro* and *in vivo* (Liu et al., 2015). Polygalacic acid (PA) significantly improves cholinergic system reactivity, as indicated by decreased AChE activity, increased choline ChAT activity, and elevated ACh levels in the hippocampus and frontal cortex. PA also ameliorates neuroinflammation and oxidative stress in mice (Guo et al., 2016). Although *P. tenuifolia* has appeared in many classic prescriptions to treat VD, its effective ingredients have rarely been studied in anti-dementia treatment. The main focus has been on the study of saponins, and the other effective ingredients, such as ketones, have not been adequately studied.

## Prospects and Conclusion

TCM theory, which is clearly distinct from Western medicine, is based on the equilibrium of Yin and Yang and the Five Elements theory. TCM has been in use for thousands of years, but there are certain limitations to its research and clinical applications: 1) TCM contains thousands of compounds that are combinations of multiple pharmacological components that can regulate cell function, so understanding the exact mechanism of drug activity is very challenging. 2) Different compounds in TCM formulae may compete with each other in signaling pathways (inhibition or activation of the same signaling pathways) or targets (receptor agonists or antagonists) to mask the underlying activity. 3) When a desired pharmacological activity has been determined, it is difficult to know which compound is responsible for the activity. Therefore, when multiple components of TCM interact, the specific mechanism of single drug and individual differences of the human body are uncertain, the study of the optimal dose, dose-response relationship, half-life, and treatment of related adverse reactions can provide the basis for the large-scale clinical application of TCM. High content screening offers a new technological means for the studying the effects of TCM, which can elucidate TCM’s role in cells as a whole, and can be used for the screening of multiple components of TCM while allowing identification of effective components (Wang et al., 2019c). It also aids in the investigation of TCM's mechanism of action from different levels and multiple targets. In addition, the rapid development of omics methods and pharmacological network analysis tools has made it easier to unravel the mysteries of TCM (Zhang et al., 2017; Guo et al., 2020).

Although there have been many basic and clinical based studies about TCM’s effect on VD in recent years, clinical studies generally have had a relatively short observation period, small sample size, with a lack of prospective evidence for large-scale and long-term follow-up. The development of VD is a slow and long-term process. Hence, the clinical efficacy of using TCM to treat VD in the short term needs to be further evaluated. TCM has a unique overall concept and a theoretical system of differentiating syndromes and treatments. Therefore, future studies should focus on development of different VD animal models and administration of TCM preparations to prove their effectiveness in VD. The existing TCM prescriptions to treat VD are very broad, and there is a lack of unified TCM prescriptions with clear clinical value for VD. Therefore, studying the effective ingredients of “single medicines” in various prescriptions will be of great practical significance and the main research direction of TCM for long-term treatment for VD.

## References

[B1] Cao Y. Gou Z. Du Y. Fan Y. Liang L. Yan Y. (2016). Glutamatergic and central Cholinergic Dysfunction in the CA1, CA2 and CA3 fields on Spatial Learning and Memory in Chronic Cerebral Ischemia-Induced Vascular Dementia of Rats. Neurosci. Lett. 620, 169–176. 10.1016/j.neulet.2016.03.039 27040427

[B2] Chan E. S. Bautista D. T. Zhu Y. You Y. Long J. T. Li W. (2018). Traditional Chinese Herbal Medicine for Vascular Dementia. Cochrane Database Syst. Rev. 12, Cd010284. 10.1002/14651858.CD010284.pub2 30520514 PMC6516869

[B3] Chen Y. Gao X. Liu Q. Zeng L. Zhang K. Mu K. (2020). Alpha-asarone Improves Cognitive Function of Aged Rats by Alleviating Neuronal Excitotoxicity via GABAA Receptors. Neuropharmacology 162, 107843. 10.1016/j.neuropharm.2019.107843 31704273

[B4] Choi E. S. Yoon J. J. Han B. H. Jeong D. H. Lee Y. J. Kang D. G. (2018). Ligustilide Attenuates Vascular Inflammation and Activates Nrf2/HO-1 Induction and, NO Synthesis in HUVECs. Phytomedicine 38, 12–23. 10.1016/j.phymed.2017.09.022 29425644

[B5] Choi R. J. Roy A. Jung H. J. Ali M. Y. Min B.-S. Park C. H. (2016). BACE1 Molecular Docking and Anti-alzheimer's Disease Activities of Ginsenosides. J. Ethnopharmacology 190, 219–230. 10.1016/j.jep.2016.06.013 27275774

[B6] Dai W. Wei C. Kong H. Jia Z. Han J. Zhang F. (2011). Effect of the Traditional Chinese Medicine Tongxinluo on Endothelial Dysfunction Rats Studied by Using Urinary Metabonomics Based on Liquid Chromatography-Mass Spectrometry. J. Pharm. Biomed. Anal. 56, 86–92. 10.1016/j.jpba.2011.04.020 21620604

[B7] Dong J. Zhao J. Lin Y. Liang H. He X. Zheng X. (2018). Exercise Improves Recognition Memory and Synaptic Plasticity in the Prefrontal Cortex for Rats Modelling Vascular Dementia. Neurol. Res. 40, 68–77. 10.1080/01616412.2017.1398389 29126372

[B8] Dong L. Hyde A. J. Zhang A. L. Xue C. C. May B. H. (2019). Chinese Herbal Medicine for Mild Cognitive Impairment Using Montreal Cognitive Assessment: A Systematic Review. J. Altern. Complement. Med. 25, 578–592. 10.1089/acm.2018.0346 30920303

[B9] Dong L. May B. H. Feng M. Hyde A. J. Tan H. Y. Guo X. (2016). Chinese Herbal Medicine for Mild Cognitive Impairment: A Systematic Review and Meta-Analysis of Cognitive Outcomes. Phytother. Res. 30, 1592–1604. 10.1002/ptr.5679 27416935

[B10] Du S.-Q. Wang X.-R. Zhu W. Ye Y. Yang J.-W. Ma S.-M. (2018). Acupuncture Inhibits TXNIP-Associated Oxidative Stress and Inflammation to Attenuate Cognitive Impairment in Vascular Dementia Rats. CNS Neurosci. Ther. 24, 39–46. 10.1111/cns.12773 29110407 PMC6489958

[B11] Ecay-Torres M. Estanga A. Tainta M. Izagirre A. Garcia-Sebastian M. Villanua J. (2018). Increased CAIDE Dementia Risk, Cognition, CSF Biomarkers, and Vascular burden in Healthy Adults. Neurology 91, e217–e226. 10.1212/wnl.0000000000005824 29898969

[B12] Escandon A. Al-Hammadi N. Galvin J. E. (2010). Effect of Cognitive Fluctuation on Neuropsychological Performance in Aging and Dementia. Neurology 74, 210–217. 10.1212/wnl.0b013e3181ca017d 20083796 PMC2809035

[B13] Fan L. H. Wang K. Z. Cheng B. Wang C. S. Dang X. Q. (2006). Anti-apoptotic and Neuroprotective Effects of Tetramethylpyrazine Following Spinal Cord Ischemia in Rabbits. BMC Neurosci. 7, 48. 10.1186/1471-2202-7-48 16774675 PMC1534051

[B14] Faraco G. Hochrainer K. Segarra S. G. Schaeffer S. Santisteban M. M. Menon A. (2019). Dietary Salt Promotes Cognitive Impairment through Tau Phosphorylation. Nature 574, 686–690. 10.1038/s41586-019-1688-z 31645758 PMC7380655

[B15] Fei Y.-L. Lv H.-J. Li Y.-B. Liu J. Qian Y.-H. Yang W.-N. (2017). Tongxinluo Improves Cognition by Decreasing β-amyloid in Spontaneous Hypertensive Rats. Brain Res. 1663, 151–160. 10.1016/j.brainres.2017.03.005 28274609

[B16] Fu J. Wang Z. Huang L. Zheng S. Wang D. Chen S. (2014). Review of the Botanical Characteristics, Phytochemistry, and Pharmacology ofAstragalus membranaceus(Huangqi). Phytother. Res. 28, 1275–1283. 10.1002/ptr.5188 25087616

[B17] Ge C.-L. Wang X.-M. Huang Z.-G. Xia Q. Wang N. Xu D.-J. (2015). Tongqiao Huoxue Decoction Ameliorates Learning and Memory Defects in Rats with Vascular Dementia by Up-Regulating the Ca 2+ -CaMKII-CREB Pathway. Chin. J. Nat. Medicines 13, 823–830. 10.1016/s1875-5364(15)30086-8 26614457

[B18] Guo C. Shen J. Meng Z. Yang X. Li F. (2016). Neuroprotective Effects of Polygalacic Acid on Scopolamine-Induced Memory Deficits in Mice. Phytomedicine 23, 149–155. 10.1016/j.phymed.2015.12.009 26926176

[B19] Guo R. Luo X. Liu J. Liu L. Wang X. Lu H. (2020). Omics Strategies Decipher Therapeutic Discoveries of Traditional Chinese Medicine against Different Diseases at Multiple Layers Molecular-Level. Pharmacol. Res. 152, 104627. 10.1016/j.phrs.2020.104627 31904505

[B20] He Y. Ruganzu J. B. Lin C. Ding B. Zheng Q. Wu X. (2020). Tanshinone IIA Ameliorates Cognitive Deficits by Inhibiting Endoplasmic Reticulum Stress-Induced Apoptosis in APP/PS1 Transgenic Mice. Neurochem. Int. 133, 104610. 10.1016/j.neuint.2019.104610 31778727

[B21] Huang K. Shen L. Niu T. Zhao Y. Fu J. Cao Y. (2019). Naomaitai Ameliorated Brain Damage in Rats with Vascular Dementia by PI3K/PDK1/AKT Signaling Pathway. Evid. Based Complement. Alternat Med. 2019, 2702068. 10.1155/2019/2702068 30867669 PMC6379870

[B22] Huang Y. C. Tsay H. J. Lu M. K. Lin C. H. Yeh C. W. Liu H. K. (2017). Astragalus Membranaceus-Polysaccharides Ameliorates Obesity, Hepatic Steatosis, Neuroinflammation and Cognition Impairment without Affecting Amyloid Deposition in Metabolically Stressed APPswe/PS1dE9 Mice. Int. J. Mol. Sci. 18. 10.3390/ijms18122746 PMC5751345 29258283

[B23] Hugo J. Ganguli M. (2014). Dementia and Cognitive Impairment. Clin. Geriatr. Med. 30, 421–442. 10.1016/j.cger.2014.04.001 25037289 PMC4104432

[B24] Huo T. Jia Y. Yin C. Luo X. Zhao J. Wang Z. (2019). Iron Dysregulation in Vascular Dementia: Focused on the AMPK/autophagy Pathway. Brain Res. Bull. 153, 305–313. 10.1016/j.brainresbull.2019.09.006 31542426

[B25] Ikram M. A. Bersano A. Manso-Calderón R. Jia J. P. Schmidt H. Middleton L. (2017). Genetics of Vascular Dementia - Review from the ICVD Working Group. BMC Med. 15, 48. 10.1186/s12916-017-0813-9 28260527 PMC5338082

[B26] Iwasaki K. Takasaki K. Nogami A. Kubota K. Katsurabayashi S. Mishima K. (2012). Pharmacological Studies for the Ameliorative Effects of Yokukansan on Memory Deficits and Abnormal Behavior in an Animal Model of Dementia. Folia Pharmacol. Jpn. 140, 66–70. 10.1254/fpj.140.66 22878569

[B27] Ji H.-J. Hu J.-F. Wang Y.-H. Chen X.-Y. Zhou R. Chen N.-H. (2010). Osthole Improves Chronic Cerebral Hypoperfusion Induced Cognitive Deficits and Neuronal Damage in hippocampus. Eur. J. Pharmacol. 636, 96–101. 10.1016/j.ejphar.2010.03.038 20362569

[B28] Li C. W. Deng M. Z. Gao Z. J. Dang Y. Y. Zheng G. D. Yang X. J. (2020). Effects of Compound K, a Metabolite of Ginsenosides, on Memory and Cognitive Dysfunction in Db/db Mice Involve the Inhibition of ER Stress and the NLRP3 Inflammasome Pathway. Food Funct. 11 (5), 4416–4427. 10.1039/c9fo02602a 32374299

[B29] Li H. B. Liang W. B. Zhou L. (2020). The Experimental Research on Neuroplasticity in Rats' hippocampus Subjected to Chronic Cerebral Hypoperfusion and Interfered by Modified Dioscorea Pills. Heliyon 6, e02897. 10.1016/j.heliyon.2019.e02897 31909235 PMC6938820

[B30] Li J. Geng J. Gan Y. Qiu D. W. (2008). [Research on Optimization of Forming Techniques for Compound Tall Gastrodia Tuber Drop Pills by Uniform Design Method]. Zhong Yao Cai 31, 902–904. 18998577

[B31] Li J. Ma Y. Y. Liu B. Mao W. J. Zhang J. X. Li S. Y. (2016). [Effect of Yangxue Qingnao Granule on the Expression of CD11b in CA1 Region of Hippocampus of Vascular Dementia Rats]. Zhongguo Zhong Xi Yi Jie He Za Zhi 36, 619–623. 27386658

[B32] Li J. Xu L. Sang R. Yu Y. Ge B. Zhang X. (2018). Immunomodulatory and Anti-inflammatory Effects of Total Flavonoids of Astragalus by Regulating NF-Κb and MAPK Signalling Pathways in RAW 264.7 Macrophages. Pharmazie 73, 589–593. 10.1691/ph.2018.8633 30223923

[B33] Li L. Chen H. Shen A. Li Q. Chen Y. Chu J. (2019). Ligustrazine Inhibits Platelet Activation via Suppression of the Akt Pathway. Int. J. Mol. Med. 43, 575–582. 10.3892/ijmm.2018.3970 30387814

[B34] Li W. Z. Li W. P. Yin Y. Y. (2007). [Effects of AST and ASI on Metabolism of Free Radical in Senescent Rats Treated by HC]. Zhongguo Zhong Yao Za Zhi 32, 2539–2542. 18330253

[B35] Liao J. Xia X. Wang G.-Z. Shi Y.-M. Ge J.-W. (2015). Naotaifang Extract Treatment Results in Increased Ferroportin Expression in the hippocampus of Rats Subjected to Cerebral Ischemia. Mol. Med. Rep. 11, 4047–4052. 10.3892/mmr.2015.3309 25672910 PMC4394947

[B36] Liu N. H. Huang X. F. Tian K. Y. Li M. Chen W. Zhu C. D. (2014). [Effects of Acupuncture and "Tongxinluo" Capsule on Plasma Lysophosphatidic Acid Level in Patients with Acute Cerebral Infarction]. Zhen Ci Yan Jiu 39, 487–511. 25632575

[B37] Liu X.-Q. Deng Y.-X. Dai Z. Hu T. Cai W.-W. Liu H.-F. (2020). Sodium Tanshinone IIA Sulfonate Protects against Aβ1-42-Induced Cellular Toxicity by Modulating Aβ-Degrading Enzymes in HT22 Cells. Int. J. Biol. Macromolecules 151, 47–55. 10.1016/j.ijbiomac.2020.02.040

[B38] Liu Y.-m. Li Z.-y. Hu H. Xu S.-p. Chang Q. Liao Y.-h. (2015). Tenuifolin, a Secondary Saponin from Hydrolysates of Polygalasaponins, Counteracts the Neurotoxicity Induced by Aβ25-35 Peptides *In Vitro* and *In Vivo* . Pharmacol. Biochem. Behav. 128, 14–22. 10.1016/j.pbb.2014.11.010 25444865

[B39] Liu Y. Huang X. Chen W. Chen Y. Wang N. Wu X. (2020). The Effects of Yuan-Zhi Decoction and its Active Ingredients in Both *In Vivo* and *In Vitro* Models of Chronic Cerebral Hypoperfusion by Regulating the Levels of Aβ and Autophagy. Evid. Based Complement. Alternat Med. 2020, 6807879. 10.1155/2020/6807879 32184897 PMC7060441

[B40] Mazzio E. A. Bauer D. Mendonca P. Taka E. Soliman K. F. A. (2017). Natural Product HTP Screening for Attenuation of Cytokine-Induced Neutrophil Chemo Attractants (CINCs) and NO2− in LPS/IFNγ Activated Glioma Cells. J. Neuroimmunology 302, 10–19. 10.1016/j.jneuroim.2016.11.012 27956075 PMC5201440

[B41] Mulugeta E. Molina-Holgado F. Elliott M. S. Hortobagyi T. Perry R. Kalaria R. N. (2008). Inflammatory Mediators in the Frontal Lobe of Patients with Mixed and Vascular Dementia. Dement Geriatr. Cogn. Disord. 25, 278–286. 10.1159/000118633 18303264

[B42] Park H.-J. Kim H. Y. Yoon K.-H. Kim K. S. Shim I. (2009). The Effects of Astragalus Membranaceus on Repeated Restraint Stress-Induced Biochemical and Behavioral Responses. Korean J. Physiol. Pharmacol. 13, 315–319. 10.4196/kjpp.2009.13.4.315 19885016 PMC2766712

[B43] Pinter D. Enzinger C. Fazekas F. (2015). Cerebral Small Vessel Disease, Cognitive reserve and Cognitive Dysfunction. J. Neurol. 262, 2411–2419. 10.1007/s00415-015-7776-6 25976029

[B44] Qian Y. Qian F. Zhang W. Zhao L. Shen M. Ding C. (2019). Shengjiang Powder Ameliorates Myocardial Injury in Septic Rats by Downregulating the Phosphorylation of P38-MAPK. J. Biosci. 44. 10.1007/s12038-019-9857-7 31180053

[B45] Quick S. Moss J. Rajani R. M. Williams A. (2021). A Vessel for Change: Endothelial Dysfunction in Cerebral Small Vessel Disease. Trends Neurosciences 44, 289–305. 10.1016/j.tins.2020.11.003 33308877

[B46] Raz L. Bhaskar K. Weaver J. Marini S. Zhang Q. Thompson J. F. (2019). Hypoxia Promotes Tau Hyperphosphorylation with Associated Neuropathology in Vascular Dysfunction. Neurobiol. Dis. 126, 124–136. 10.1016/j.nbd.2018.07.009 30010004 PMC6347559

[B47] Román G. C. Erkinjuntti T. Wallin A. Pantoni L. Chui H. C. (2002). Subcortical Ischaemic Vascular Dementia. Lancet Neurol. 1, 426–436. 10.1016/s1474-4422(02)00190-4 12849365

[B48] Saki G. Eidi A. Mortazavi P. Panahi N. Vahdati A. (2020). Effect of β-asarone in normal and β-amyloid-induced Alzheimeric Rats. aoms 16, 699–706. 10.5114/aoms.2020.94659 PMC7212238 32399120

[B49] Sun Z.-K. Ma X.-R. Jia Y.-J. Liu Y.-R. Zhang J.-W. Zhang B.-A. (2014). Effects of Resveratrol on Apoptosis in a Rat Model of Vascular Dementia. Exp. Ther. Med. 7, 843–848. 10.3892/etm.2014.1542 24660032 PMC3961111

[B50] Tan F. Fu W. Cheng N. Meng D. Gu Y. (2015). Ligustrazine Reduces Blood-Brain Barrier Permeability in a Rat Model of Focal Cerebral Ischemia and Reperfusion. Exp. Ther. Med. 9, 1757–1762. 10.3892/etm.2015.2365 26136889 PMC4471771

[B51] Tarkowski E. Wallin A. Regland B. Blennow K. Tarkowski A. (2001). Local and Systemic GM-CSF Increase in Alzheimer's Disease and Vascular Dementia. Acta Neurol. Scand. 103, 166–174. 10.1034/j.1600-0404.2001.103003166.x 11240564

[B52] Téglás T. Németh Z. Koller Á. Van Der Zee E. A. Luiten P. G. M. Nyakas C. (2019). Effects of Long-Term Moderate Intensity Exercise on Cognitive Behaviors and Cholinergic Forebrain in the Aging Rat. Neuroscience 411, 65–75. 10.1016/j.neuroscience.2019.05.037 31146009

[B53] Tsai C.-F. Thomas B. Sudlow C. L. M. (2013). Epidemiology of Stroke and its Subtypes in Chinese vs white Populations: a Systematic Review. Neurology 81, 264–272. 10.1212/wnl.0b013e31829bfde3 23858408 PMC3770160

[B54] Viswanathan A. Rocca W. A. Tzourio C. (2009). Vascular Risk Factors and Dementia: How to Move Forward?. Neurology 72, 368–374. 10.1212/01.wnl.0000341271.90478.8e 19171835 PMC2677504

[B55] Voet S. Srinivasan S. Lamkanfi M. Van Loo G. (2019). Inflammasomes in Neuroinflammatory and Neurodegenerative Diseases. EMBO Mol. Med. 11. 10.15252/emmm.201810248 PMC6554670 31015277

[B56] Wang D.-P. Yin H. Lin Q. Fang S.-P. Shen J.-H. Wu Y.-F. (2019). Andrographolide Enhances Hippocampal BDNF Signaling and Suppresses Neuronal Apoptosis, Astroglial Activation, Neuroinflammation, and Spatial Memory Deficits in a Rat Model of Chronic Cerebral Hypoperfusion. Naunyn-schmiedeberg's Arch. Pharmacol. 392, 1277–1284. 10.1007/s00210-019-01672-9 31187188

[B57] Wang H.-c. Liu N.-y. Zhang S. Yang Y. Wang Z.-Y. Wei Y. (2020). Clinical Experience in Treatment of Alzheimer's Disease with Jiannao Yizhi Formula (健脑益智方) and Routine Western Medicine. Chin. J. Integr. Med. 26, 212–218. 10.1007/s11655-019-2718-2 32180150

[B58] Wang H. Liu N. Wei Y. Pei H. Liu M. Diao X. (2019). Efficacy and Safety of Shenmayizhi Decoction as an Adjuvant Treatment for Vascular Dementia: Study Protocol for a Randomized Controlled Trial. Medicine (Baltimore) 98, e18326. 10.1097/md.0000000000018326 31852125 PMC6922576

[B59] Wang J. He X. Chen W. Zhang N. Guo J. Liu J. (2020). Tanshinone IIA Protects Mice against Atherosclerotic Injury by Activating the TGF-β/PI3K/Akt/eNOS Pathway. Coron. Artery Dis. 31, 385–392. 10.1097/mca.0000000000000835 31842027 PMC7192539

[B60] Wang J. Wu M. Y. Tan J. Q. Li M. Lu J. H. (2019). High Content Screening for Drug Discovery from Traditional Chinese Medicine. Chin. Med. 14, 5. 10.1186/s13020-019-0228-y 30858873 PMC6394041

[B61] Wang X. Zhang D. Song W. Cai C. F. Zhou Z. Fu Q. (2020). Neuroprotective Effects of the Aerial Parts of Polygala Tenuifolia Willd Extract on Scopolamine-Induced Learning and Memory Impairments in Mice. Biomed. Rep. 13, 37. 10.3892/br.2020.1344 PMC7453304 32874571

[B62] Wen W. Sachdev P. S. Li J. J. Chen X. Anstey K. J. (2009). White Matter Hyperintensities in the Forties: Their Prevalence and Topography in an Epidemiological Sample Aged 44-48. Hum. Brain Mapp. 30, 1155–1167. 10.1002/hbm.20586 18465744 PMC6870596

[B63] Wu H. Y. Li H. L. Yang C. S. Wang H. P. Gu J. (2015). [Effect of Guiqicongzhi Decoction on Expression of HSP27 and HSP70 in Brain Tissue of VD Model Rats]. Zhong Yao Cai 38, 2578–2582. 27352542

[B64] Wu J. Lu A. D. Zhang L. P. Zuo Y. X. Jia Y. P. (2019). [Study of Clinical Outcome and Prognosis in Pediatric Core Binding Factor-Acute Myeloid Leukemia]. Zhonghua Xue Ye Xue Za Zhi 40, 52–57. 10.3760/cma.j.issn.0253-2727.2019.01.010 30704229 PMC7351698

[B65] Wu L. Zhao Q.-S. Li T.-W. Li H.-Y. Wang Q.-B. Bi X.-Y. (2015). Yifei Xuanfei Jiangzhuo Formula, a Chinese Herbal Decoction, Improves Memory Impairment through Inhibiting Apoptosis and Enhancing PKA/CREB Signal Transduction in Rats with Cerebral Ischemia/reperfusion. Mol. Med. Rep. 12, 4273–4283. 10.3892/mmr.2015.3962 26094797 PMC4526035

[B66] Wu Q. Cao Y. Liu M. Liu F. Brantner A. H. Yang Y. (2019). Traditional Chinese Medicine Shenmayizhi Decoction Ameliorates Memory and Cognitive Impairment Induced by Scopolamine via Preventing Hippocampal Cholinergic Dysfunction in Rats. Ndt Vol. 15, 3167–3176. 10.2147/ndt.s214976 PMC6858809 31814724

[B67] Wu X.-l. An P. Ye B.-y. Shi X.-m. Sun W.-s. Fu R.-g. (2013). Qufeng Tongluo Prescription (祛风通络方) Inhibits Mesangial Cell Proliferation and Promotes Apoptosis through Regulating Cell Cycle Progression. Chin. J. Integr. Med. 19, 927–934. 10.1007/s11655-013-1655-8 24307313

[B68] Xiong X. Wang P. Zhang Y. Li X. (2015). Effects of Traditional Chinese Patent Medicine on Essential Hypertension. Medicine (Baltimore) 94, e442. 10.1097/md.0000000000000442 25654379 PMC4602722

[B69] Xu J. Qi Q. Lv P. Dong Y. Jiang X. Liu Z. (2019). Oxiracetam Ameliorates Cognitive Deficits in Vascular Dementia Rats by Regulating the Expression of Neuronal Apoptosis/autophagy-Related Genes Associated with the Activation of the Akt/mTOR Signaling Pathway. Braz. J. Med. Biol. Res. 52, e8371. 10.1590/1414-431x20198371 31721903 PMC6853072

[B70] Yang P. Dong K. L. Zeng W. Y. (2006). [Effect of Yizhi Jiannao Granule on the Behavior and Neuron Apoptosis in SAMP/8 Mice]. Zhong Nan Da Xue Xue Bao Yi Xue Ban 31, 56–59. 16562676

[B71] Yin C. H. Bi D. P. Du M. (2010). [Effect of Tongxinluo Capsule on Platelet Aggregation Function in Patients with Aspirin Resistance]. Zhongguo Zhong Xi Yi Jie He Za Zhi 30, 380–382. 20669674

[B72] Zhang B. L. Wang Y. Y. Chen R. X. (2002). [Clinical Randomized Double-Blinded Study on Treatment of Vascular Dementia by Jiannao Yizhi Granule]. Zhongguo Zhong Xi Yi Jie He Za Zhi 22, 577–580. 12572375

[B73] Zhang D. P. Lu X. Y. He S. C. Li W. Y. Ao R. Leung F. C. Y. (2020). Sodium Tanshinone IIA Sulfonate Protects against Aβ‐induced Cell Toxicity through Regulating Aβ Process. J. Cel Mol Med. 24, 3328–3335. 10.1111/jcmm.15006 PMC7131914 31989795

[B74] Zhang R. Z. Yu S. J. Bai H. Ning K. (2017). TCM-mesh: The Database and Analytical System for Network Pharmacology Analysis for TCM Preparations. Sci. Rep. 7, 2821. 10.1038/s41598-017-03039-7 28588237 PMC5460194

[B75] Zhang Y. Yang X. Wang S. Song S. (2019). Ginsenoside Rg3 Prevents Cognitive Impairment by Improving Mitochondrial Dysfunction in the Rat Model of Alzheimer's Disease. J. Agric. Food Chem. 67, 10048–10058. 10.1021/acs.jafc.9b03793 31422666

[B76] Zhao T. Fu Y. Sun H. Liu X. (2018). Ligustrazine Suppresses Neuron Apoptosis via the Bax/Bcl-2 and Caspase-3 Pathway in PC12 Cells and in Rats with Vascular Dementia. IUBMB Life 70, 60–70. 10.1002/iub.1704 29247598

[B77] Zhu Z. Zhang L. Cui Y. Li M. Ren R. Li G. (2020). Functional Compensation and Mechanism of Choline Acetyltransferase in the Treatment of Cognitive Deficits in Aged Dementia Mice. Neuroscience 442, 41–53. 10.1016/j.neuroscience.2020.05.016 32497760

[B78] Zong W. Zeng X. Chen S. Chen L. Zhou L. Wang X. (2019). Ginsenoside Compound K Attenuates Cognitive Deficits in Vascular Dementia Rats by Reducing the Aβ Deposition. J. Pharmacol. Sci. 139, 223–230. 10.1016/j.jphs.2019.01.013 30799178

